# *In vivo* biofilm formation on stainless steel bonded retainers during different oral health-care regimens

**DOI:** 10.1038/ijos.2014.69

**Published:** 2015-01-09

**Authors:** Marije A Jongsma, Henny C van der Mei, Jelly Atema-Smit, Henk J Busscher, Yijin Ren

**Affiliations:** 1 Department of Orthodontics, University of Groningen and University Medical Centre, Groningen, The Netherlands; 2 Department of Biomedical Engineering, University of Groningen and University Medical Centre, Groningen, The Netherlands

**Keywords:** antimicrobials, biofilm, bonded retention wires, mouthrinse, orthodontics

## Abstract

Retention wires permanently bonded to the anterior teeth are used after orthodontic treatment to prevent the teeth from relapsing to pre-treatment positions. A disadvantage of bonded retainers is biofilm accumulation on the wires, which produces a higher incidence of gingival recession, increased pocket depth and bleeding on probing. This study compares *in vivo* biofilm formation on single-strand and multi-strand retention wires with different oral health-care regimens. Two-centimetre wires were placed in brackets that were bonded to the buccal side of the first molars and second premolars in the upper arches of 22 volunteers. Volunteers used a selected toothpaste with or without the additional use of a mouthrinse containing essential oils. Brushing was performed manually. Regimens were maintained for 1 week, after which the wires were removed and the oral biofilm was collected to quantify the number of organisms and their viability, determine the microbial composition and visualize the bacteria by electron microscopy. A 6-week washout period was employed between regimens. Biofilm formation was reduced on single-strand wires compared with multi-strand wires; bacteria were observed to adhere between the strands. The use of antibacterial toothpastes marginally reduced the amount of biofilm on both wire types, but significantly reduced the viability of the biofilm organisms. Additional use of the mouthrinse did not result in significant changes in biofilm amount or viability. However, major shifts in biofilm composition were induced by combining a stannous fluoride- or triclosan-containing toothpaste with the mouthrinse. These shifts can be tentatively attributed to small changes in bacterial cell surface hydrophobicity after the adsorption of the toothpaste components, which stimulate bacterial adhesion to the hydrophobic oil, as illustrated for a *Streptococcus mutans* strain.

## Introduction

A major challenge in orthodontics is the retention of treatment results after the removal of orthodontic appliances. Long-term results of orthodontic treatment indicate a relapse of crowding without the use of retention devices.^1^ To prevent relapse, permanent retention wires are often bonded to the anterior teeth.^2^ Different types of retention wires can be used, including single-strand retainers bonded only to the canines or multi-strand retainers that are bonded to all six anterior teeth.^3,4^ A disadvantage of retention wires is the accumulation of biofilm and calculus along the wires, which may cause a higher incidence of gingival recession, increased pocket depth and bleeding on probing.^5,6^

*In vitro* studies have indicated that wire morphology influences the number of viable organisms in the biofilm on retention wires.^7^ Biofilms pre-formed on single-strand wires yielded less viable organisms than biofilms formed on multi-strand wires after a single exposure to a NaF-sodium lauryl sulphate-containing toothpaste slurry and an essential oil-containing mouthrinse, demonstrating that biofilms on multi-strand wires are less susceptible to oral antimicrobials than biofilms on single-strand wires. The biofilm mode of growth protects its inhabitants against the penetration of antimicrobial agents,^8^ and this effect may be enhanced when the biofilm forms in the crevices and niches of retention wires.^9^ However, it is unclear how these differences in the susceptibility of oral biofilms pre-formed on different wire morphologies *in vitro* correspond to biofilm formation *in vivo* during the use of antibacterial health-care products, such as toothpastes or mouthrinses with antibacterial effects.

In most of the population, not all biofilm is removed by mechanical means, and despite the difficulty of antimicrobial penetration of a biofilm, oral antimicrobials generally have a favourable effect on biofilm inhibition *in vivo*.^10,11,12,13^ The biofilm left behind after brushing, both dead and alive, can play an important role in improving antimicrobial action because this biofilm material can absorb antimicrobials, which are then released over time in antimicrobially effective amounts.^14^ However, the clinical relevance of this phenomenon for producing measurable effects on biofilm formation is unclear.

The aim of this study was to compare biofilm formation *in vivo* on both single-strand and multi-strand retention wires during different oral health-care regimens and to determine whether the use of oral antimicrobials influences biofilm composition. The regimens included manual brushing. Two toothpastes with antibacterial claims^15^ that contained either stannous fluoride or triclosan or a fluoridated toothpaste without antibacterial claims were used. Toothpastes were employed with or without the additional use of a mouthrinse containing essential oils.^12^

## Materials and methods

### Retainers, volunteers and inclusion criteria

Two different types of retainers were evaluated in this study, a single-strand wire (Forestanit® Forestadent, Pforzheim, Germany) and a multi-strand wire (Quadcat® PG Supply, Avon, CT, USA). Brackets (SPEED System Orthodontics, Cambridge, Canada) were bonded to the buccal side of the first molar and the second premolar in the upper arch of 22 healthy volunteers in agreement with the rules set by the Ethics Committee at the University Medical Centre Groningen (letter, 23 June 2011). The length of wire between the brackets was 2 cm. The wires were sterilized in 70% ethanol before use and were maintained *in situ* for 1 week, during which the volunteers were instructed to brush for 2 min twice a day with a manual toothbrush (Lactona iQ X-Soft; Lactona Europe B.V., Bergen op Zoom, The Netherlands) and a toothpaste with antibacterial claims (Oral-B Pro Expert® Procter & Gamble, Cincinnati, OH, USA, or Colgate Total® Colgate-Palmolive Company, Piscataway, NJ, USA) or a toothpaste without antibacterial claims that contained only NaF-sodium lauryl sulphate (Prodent Softmint® Sara Lee Household & Bodycare, Exton, PA, USA). Toothpaste was used either without additional oral hygiene measures or in combination with an essential oil-containing mouthrinse (Cool Mint Listerine® Pfizer Consumer Healthcare, Morris Plains, NJ, USA).

Between regimens, a washout period of 6 weeks was applied during which only the NaF-sodium lauryl sulphate-containing toothpaste without antibacterial claims was used. The duration of the washout period was based on the results of a pilot study that indicated that the composition of the oral biofilm returns to baseline values within 5 weeks after terminating the use of an antibacterial toothpaste.

**Figure 1:**
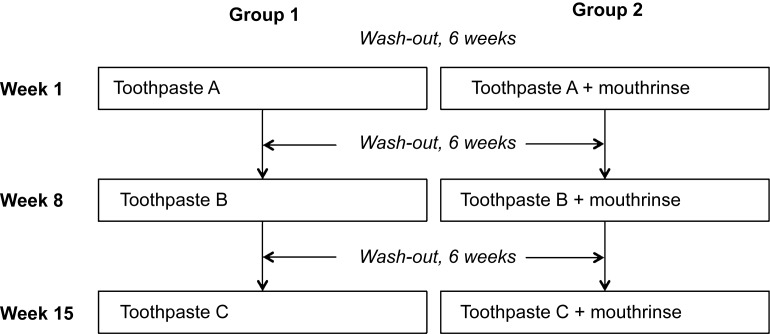
**Schematic description of the two experimental groups.** Each group consisting of 11 volunteers. Toothpastes were randomly assigned and included the following: toothpaste without antibacterial claims (Prodent Softmint; Sara Lee Household & Bodycare, Exton, PA, USA); stannous fluoride-containing toothpaste (Oral-B Pro Expert; Procter & Gamble, Cincinnati, OH, USA); triclosan-containing toothpaste (Colgate Total; Colgate-Palmolive Company, Piscataway, NJ, USA). The mouthrinse was Cool Mint Listerine® (Pfizer Consumer Healthcare, Morris Plains, NJ, USA).

Volunteers were included in the study if they had healthy and complete dentition, no bleeding upon probing, did not use any medication and did not smoke. All volunteers provided written informed consent. After inclusion, volunteers were randomly assigned to two groups. The first group successively used three different types of toothpaste, and the second group combined the same toothpastes with an antimicrobial mouthrinse (Figure 1).

Regimens were maintained for 1 week, after which the wires were removed and the oral biofilm was collected from the buccal enamel surfaces for reference using a cotton swab. Unstimulated salivary samples were also taken. The wires, collected enamel biofilms and salivary samples were stored in an Eppendorf tube containing 1.0 mL of filter-sterilized reduced transport fluid.^16^ Saliva samples were stored on ice.

To enumerate organisms, retention wires with adherent biofilm and cotton swabs with oral biofilm collected from enamel were both stored in Eppendorf tubes containing 1.0 mL of filter-sterilized reduced transport fluid; the saliva samples were separately sonicated three times for 10 s at 30-s intervals in ice-chilled water to disperse the bacteria. The bacteria were then enumerated in a Bürker-Türk counting chamber. In addition, the percentage viability of the biofilms was evaluated after live/dead staining (BacLight^TM^; Invitrogen, Breda, The Netherlands) of dispersed biofilms. Live/dead stain was prepared by adding 3 µL of SYTO®9/propidium iodide (1∶3) to 1 mL of sterile, demineralized water. Then, 15 µL of the stain was added to 10 µL of the undiluted biofilm dispersion. After a 15-min incubation in the dark, the numbers of live and dead bacteria were counted using a fluorescence microscope (Leica DM4000B; Leica Microsystems Heidelberg GmbH, Heidelberg, Germany) and expressed as percentage viability. Scanning electron micrographs of the biofilms on wires were acquired as described below.

### Denaturing gradient gel electrophoresis analysis of *in vivo* biofilms

All samples of the *in vivo* formed biofilms and the saliva were stored at −80 °C until comparison of their microbial composition by polymerase chain reaction (PCR)-denaturing gradient gel electrophoresis (DGGE). To extract DNA, the samples were thawed, centrifuged for 5 min at 13 000*g* (Eppendorf Centrifuge 5415D; Eppendorf Instruments, Hamburg, Germany) and subsequently washed and vortexed with 200 µL of TE-buffer (10 mmol⋅L^−1^ tris(hydroxymethyl)aminomethane (Tris)-HCl, 1 mmol⋅L^−1^ ethylenediamine tetraacetic acid pH 7.4), followed by centrifugation for 5 min at 13 000*g*. Next, the supernatant was removed, and the pellet was heated in a microwave (500 W, 5 min) and then suspended in 50 µL TE-buffer, vortexed and placed on ice. The quality and quantity of the DNA samples were measured using a NanoDrop® spectrophotometer (ND-1000; NanoDrop Technologies, Wilmington, DE, USA) at 230 nm. The final concentration of each DNA sample was adjusted to 100 ng of DNA for PCR amplifications.

PCR was performed with a Tgradient thermocycler (Bio-Rad I-cycler; GENOtronics BV, Landgraaf, The Netherlands). To amplify the 16S rRNA gene, the following bacterial primers were used: F357-GC (forward primer, 5′-GC clamp-TACGGGAGGCAGCAG-3′)^17^ containing a GC clamp (5′-CGCCCGCCGCGCCCCGCGCCCGGCCCGCCGCCCCCGCCCC-3′)^18^ for use in DGGE, and R-518 (reverse primer, 5′-ATTACCGCGGCTGCTGG-3′).^19^ Each 25-μL PCR mixture contained 12.5 µL of PCR Master Mix (0.05 units per μL Taq DNA polymerase in reaction buffer, 4 mmol⋅L^−1^ MgCl_2_, 0.4 mmol⋅L^−1^ dATP, 0.4 mmol⋅L^−1^ dCTP, 0.4 mmol⋅L^−1^ dGTP, 0.6 mmol⋅L^−1^ dTTP (Fermentas Life Sciences, Burlington, ON, Canada)), 1 µL each of forward and reverse primers (1 µmol⋅L^−1^) and 100 ng of DNA (in a volume of 10.5 µL). The temperature profile included an initial denaturing step at 94 °C for 5 min, followed by a denaturing step at 94 °C for 45 s, a primer annealing step at 58 °C for 45 s, an extension step at 72 °C for 1 min and a final extension step of 72 °C for 5 min. PCR products were analysed by electrophoresis on a 2.0% agarose gel containing 0.5 µg⋅mL^−1^ ethidium bromide.

DGGE of the PCR products generated with the F357-GC/R-518 primer set was performed as described by Muyzer *et al*.^20^ using a PhorU system (INGENY, Goes, The Netherlands). The PCR products were applied to an 0.08 g⋅mL^−1^ polyacrylamide gel in 0.5× TAE buffer (20 mmol⋅L^−1^ Tris acetate, 10 mmol⋅L^−1^ sodium acetate, 0.5 mmol⋅L^−1^ EDTA, pH 8.3). The denaturing gradient consisted of 30%–80% denaturant (100% denaturant equals 7 mol⋅L^−1^ urea and 37% formamide). Gels were poured using a gradient mixer. A 10-mL stacking gel without denaturant was poured on top. Electrophoresis was performed overnight at 120 V and 60 °C. Gels were stained with silver nitrate.^18^ Each DGGE gel was normalized to a marker consisting of seven reference bacterial species associated with oral health and disease^21^ and stored at 4 °C. The reference strains included *Lactobacillus *sp., *Streptococcus oralis* ATCC 35037, *Streptococcus mitis* ATCC 9811, *Streptococcus sanguinis* ATCC 10556, *Streptococcus salivarius* HB, *Streptococcus sobrinus* ATCC 33478 and *S. mutans* ATCC 10449.^14^

### Scanning electron microscopy

Biofilms on the different wires were visualized using scanning electron microscopy (SEM). The wires were fixed overnight in 2% glutaraldehyde and post-fixed for 1 h with 1% osmium tetroxide. After dehydration in a water–ethanol series, the wires were incubated in tetramethylsilane, air-dried and sputter-coated with a gold–palladium alloy, after which they were fixed on SEM-stub-holders using double-sided sticky carbon tape and visualized using a field emission SEM, type 6301F (JEOL Ltd, Tokyo, Japan) at 2 kV with a working distance of 39 mm and a small spot size.

### Statistical analysis

Data were analysed using the Statistical Package for Social Sciences (Version 16.0; SPSS Chicago, IL, USA). One-way analysis of variance was used to compare the number of bacteria and their percentage viability. A Bonferroni test was used for *post-hoc* multiple comparisons. Statistical significance was set at *P*<0.05.

DGGE gel images were converted and transferred to a microbial database using GelCompar II, version 6.1 (Applied Maths N.V, Sint-Martens-Latem, Belgium). The similarities in the bacterial compositions of the different biofilms and salivary samples were analysed using a band-based similarity coefficient and a non-weighted pair group method in which arithmetic averages were used to generate dendograms indicating similarities in composition.^22^

## Results

A slightly but significantly higher total number of bacteria were collected from the multi-strand wires compared with the single-strand wires, regardless of the oral health-care regimen applied (*P*<0.01, Table 1). The percentage viability of the bacteria adhering to the different types of wires was significantly higher for single-strand wires compared with multi-strand wires (*P<*0.05) and buccal enamel surfaces (*P<*0.001) when using a standard, fluoridated toothpaste without antibacterial claims, independent of the additional use of an essential oil-containing mouthrinse.

The use of antibacterial toothpastes without the mouthrinse had little effect on the total number of bacteria retrieved from the wires but significantly reduced their viability (*P<*0.001). The viability remained higher on the wires than on the buccal enamel surfaces. The use of a triclosan-containing toothpaste with the mouthrinse yielded the lowest number and viability of adhering bacteria on either wire.

**Figure 2:**
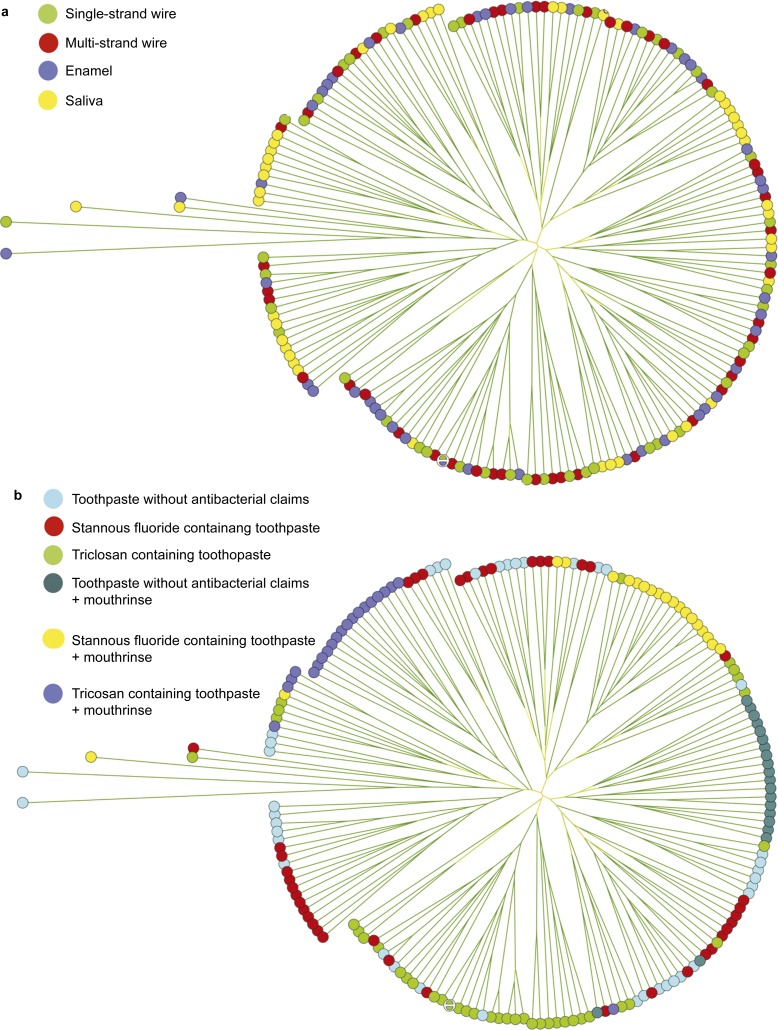
**Clustering trees describing the bacterial compositions of the microbial samples taken from the different volunteers in this study.** The corresponding circles in **a** and **b** represent the same sample. (**a**) Colours indicate different locations of microbial sampling, *i.e.*, enamel, retention wires or saliva. (**b**) Colours indicate the use of different oral health-care regimens.

The microbial compositions of the biofilms adhering to the different wires and buccal enamel surfaces and of the salivary microbiome were compared using a cluster tree (Figure 2a) combining the different oral hygiene regimens. The composition of the salivary microbiome was distinct from that of the different adhering biofilms, with a higher prevalence of *S. salivarius* and a lower prevalence of *S. mutans* (Table 2). Biofilms adhering to the wires had a higher prevalence of *Lactobacilli* and *S. sobrinus* than biofilms adhering to the buccal enamel surfaces (Table 2).

Combining the results for the different biofilms revealed an influence of the oral health-care regimen (Figure 2b). Regimens involving only the triclosan-containing toothpaste and regimens involving the use of the different individual toothpastes in combination with the mouthrinse formed clear clusters. The regimen involving only the stannous fluoride toothpaste yielded a decrease in the prevalence of lactobacilli, *S. oralis*/*S. mitis* and *S. sanguinis* compared with the toothpaste without antibacterial claims, and this decrease continued when the stannous fluoride-containing paste was used with the mouthrinse. In the latter combined regime, *S. salivarius* was also less prevalent (Table 2). The prevalence of *S. sobrinus* and *S. mutans* in the biofilms adhering to the wires and buccal enamel surfaces was not affected by the combined use of toothpaste without antibacterial claims and mouthrinse compared to toothpaste alone. The triclosan-containing toothpaste produced major increases in the prevalence of adherent *S. oralis*/*S. mitis*, *S. sanguinis* and *S. mutans*. However, the combination of the triclosan-containing toothpaste with the essential oil-containing mouthrinse resulted in the lowest prevalence of *Lactobacilli*, *S. sobrinus* and *S. mutans* among the different regimens.

**Figure 3:**
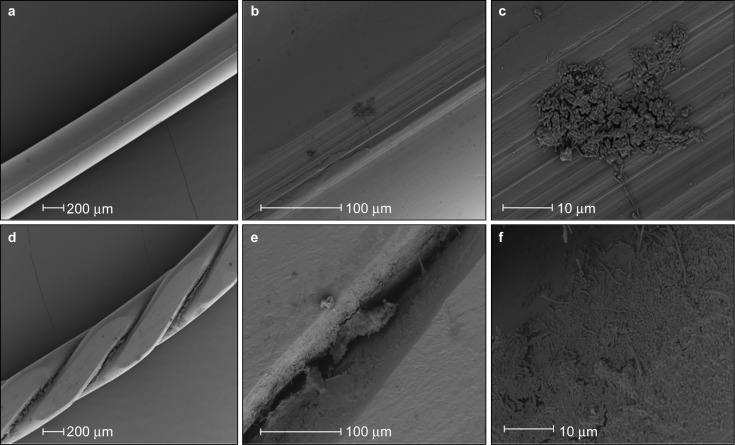
**Scanning electron micrographs of 1-week-old biofilms formed *in vivo* during use of a toothpaste without antibacterial claims.** (**a**–**c**) Single-strand wire. (**d**–**f**) Multi-strand wire.

Scanning electron micrographs (Figure 3) revealed the protected location of bacteria adhering to multi-strand wires. On the multi-strand wires, the bacteria were mostly located in the crevices between strands, whereas on the single-strand wires, the bacteria were present as a thin scattered film, attached mainly to irregularities on the wire surface.

## Discussion

Biofilm formation *in vivo* on both single-strand and multi-strand retention wires during the use of antibacterial toothpastes and a mouthrinse was evaluated. Although statistically significant differences in the numbers of bacteria adhering to the retention wires were observed for the use of different toothpastes with and without antibacterial claims alone or in combination with an essential oil-containing mouthrinse, these differences are likely too small to be of clinical significance. These results are consistent with the results of clinical studies showing that antibacterial toothpastes, including the two included in this study, reduce oral biofilm formation.^23,24^ Clinical studies have also confirmed little to no effect of the additional use of an essential oil-containing mouthrinse on oral biofilm formation.^12,25,26^ More interestingly from a clinical perspective, the use of antibacterial toothpastes reduced the percentage viability of the adhering organisms. Statistically significant but likely clinically irrelevant differences in the number of bacteria adhering to single- and multi-strand wires were also observed, but more importantly, the antibacterial regimens caused a more substantial decrease in the viability of the adhering organisms on single-strand wires compared with multi-strand wires, indicating better penetration of antimicrobials into biofilms that form on single-strand wires. These results are consistent with the higher viability of biofilms that form on single-strand wires compared with multi-strand wires during the use of a toothpaste without antibacterial claims. This increased viability is most likely due to the improved access to nutrients of bacteria adhering to single-strand wires compared with multi-strand wires.^27^

The biofilms on both types of retention wires had approximately the same microbial composition (Table 2), with some differences in the composition of the oral biofilm on enamel surfaces. The composition of adhering biofilms is very different from that of the salivary microbiome. These substratum-dependent microbial compositions confirm recent work^28^ that the surface dictates the composition of the biofilm it attracts through differential adhesion forces exerted on different strains of bacteria. The largest differences in microbial composition in biofilms adhering to retention wires and enamel surfaces were observed after regimens of antibacterial toothpastes combined with the essential oil-containing rinse. Most strikingly and of clinical importance, a regimen comprising a triclosan-containing toothpaste complemented with an essential oil-containing mouthrinse yielded a reduction in the prevalence of *S. mutans* from 30% to 5%. Other combination regimens increased the prevalence of *S. mutans* in retainer biofilms. This drastic shift in the composition of the oral microbiome in a less-cariogenic direction, *i.e.*, less *S. mutans*, by the combination of a triclosan-containing toothpaste with an essential oil-containing mouthrinse is intriguing. Oil-containing mouthrinses remove bacteria from the oral cavity through bacterial adhesion to the hydrophobic oil, which requires a certain degree of hydrophobicity of the bacterial cell surface.^29^ Moreover, certain concentrations of cationic antibacterial agents, such as cetylpyridinium chloride and chlorhexidine, promote the binding of oral microorganisms to oil droplets.^30^

Hypothetically, exposure to the non-polar triclosan^31^ could increase the hydrophobicity of the *S. mutans* cell surface, which would facilitate the removal of *S. mutans* by hydrophobic oils. To verify this hypothesis, we exposed the *S. mutans* strain used in this study to supernatants of the different toothpastes and examined its removal by a hexadecane in the so-called kinetic MATH assay,^32^ as described in Supplementary Information. The removal rate of *S. mutans* by hexadecane was low (Supplementary Figure S1), classifying the surface of *S. mutans* as slightly hydrophilic (Supplementary Table S1). Only exposure to the triclosan-containing toothpaste supernatant increased the removal of *S. mutans* by hexadecane (see also Supplementary Figure S1 and Supplementary Table S1). This finding supports our hypothesis that exposure to triclosan can increase the hydrophobicity of the *S. mutans* cell surface, facilitating its removal by oil-containing mouthrinses, and corresponds with the observation that *S. mutans* strains grown in the presence of triclosan form more extensive biofilms.^33^ However, the authors of the latter paper ruled out the effects of triclosan on streptococcal cell surface hydrophobicity, most likely because they did not use the MATH assay in its more sensitive kinetic mode as advocated by Lichtenberg *et al*.^32^ Methods of influencing the composition of oral biofilms towards a ‘healthy' composition are in the preliminary stages of development. The adsorption of toothpaste components to increase the hydrophobicity of the surfaces of selected oral pathogens and the use of hydrophobic oil-containing mouthrinses for the subsequent removal of these pathogens from the oral cavity seem to be a clinically feasible approach. The present results warrant more research into components that alter the cell surface hydrophobicity of selected oral bacterial strains.

Whether the compositional changes observed have any beneficial clinical effect remains unclear. However, the use of an antibacterial toothpaste containing sodium lauryl sulphate and stannous fluoride or triclosan increases the pH of oral biofilms and decreases their viability,^34,35^ resulting in a less cariogenic biofilm. Clearly, such changes in biofilm properties may be considered an indication of an altered microbial composition if not of a reduced prevalence of *S. mutans* and *Lactobacilli* in the biofilm. Most studies on oral biofilm composition have employed a control regimen, such as the use of a NaF-sodium lauryl sulphate-containing toothpaste with mint flavour in our study. This paste was chosen as a control because it has no antimicrobial claims; however, potential effects of this toothpaste on oral biofilm viability and composition cannot be ruled out. Both fluoride and sodium lauryl sulphate as well as mint flavouring agents have antibacterial properties,^15,36,37^ and fluoride inhibits calcium-bridging between co-adhering pairs of oral bacteria.^38^

In summary, *in vivo,* slightly less oral biofilm is formed on single-strand retention wires compared with multi-strand wires. Orthodontic patients with a fixed bonded retainer would benefit from the use of an appropriate regimen of an antibacterial toothpaste and mouthrinse based on a reduction of biofilm viability rather than biofilm formation. Appropriate regimens may increase the hydrophobicity of selected members of the oral microbiome through the adsorption of non-polar components from toothpastes to subsequently enhance their removal by oil-containing mouthrinses, resulting in less pathogenic biofilms. However, this pathway to restoring a healthy oral microbiome requires further exploration.

## Conflicts of interests

The authors declare no potential conflicts of interest with respect to authorship and/or publication of this article. The opinions and assertions contained herein are those of the authors and do not necessarily represent the views of the companies that donated the various wires or their respective employees.

## Figures and Tables

**Table 1:** The number and viability of bacteria retrieved from 1-cm retainer wires treated with the different toothpastes alone or in combination with the essential oil-containing mouthrinse

	Number of bacteria (log-units)		Live bacteria/%		
Treatments	Single-strand	Multi-strand	Single-strand	Multi-strand	Enamel
Toothpaste without antibacterial claims	7.5±0.2^a^	8.0±0.2	68±10a,d	51±19	38±14
Toothpaste without antibacterial claims+mouthrinse	7.2±0.2^a^	7.8±0.2	78±8a,d	57±12	46±11
Stannous fluoride-containing toothpaste	7.3±0.1^a^	7.8±0.3	25±8^e^	36±10^e^	20±12^e^
Stannous fluoride-containing toothpaste+mouthrinse	7.0±0.1a,b	7.5±0.3^b^	24±10^e^	32±11^e^	22±10^e^
Triclosan-containing toothpaste	7.1±0.2a,b	7.7±0.3^b^	27±8^e^	30±4^e^	17±8^e^
Triclosan-containing toothpaste+mouthrinse	6.6±0.2a,b,c	7.4±0.2^b^	23±7^e^	28±4^e^	19±4^e^

For reference, the viabilities on buccal enamel surfaces are also provided; however, for experimental reasons, no comparative data on the total numbers of adhering bacteria are provided. All data are expressed as averages±standard deviations over 11 different volunteers.

a Significantly different from multi-strand wire.

b Significantly different from a toothpaste without antibacterial claims.

c Significantly different from toothpaste only.

d Significantly different from enamel.

e Significantly different from a toothpaste without antibacterial claims, with or without the use of mouthrinse.

**Table 2:** Prevalence of marker strains in microbial samples from biofilms adhering to the different wires and buccal enamel surfaces and in the salivary microbiome for different oral health-care regimens

	Combining oral health-care regimens					
Strains	Single-strand wire	Multi-strand wire		Enamel		Saliva
*Lactobacillus*	20	25		13		22
*S. oralis/mitis*	55	56		57		65
*S. sanguinis*	63	60		65		57
*S. salivarius*	16	21		20		57
*S. sobrinus*	45	52		39		46
*S. mutans*	57	48		57		35
	**Combining biofilms adhering to wires and buccal enamel surfaces**					
**Strains**	**Toothpaste without antibacterial claims**	**Toothpaste without antibacterial claims+mouthrinse**	**Stannous fluoride-containing toothpaste**	**Stannous fluoride-containing toothpaste+mouthrinse**	**Triclosan-containing toothpaste**	**Triclosan-containing toothpaste+mouthrinse**
*Lactobacillus*	30	45	21	5	11	5
*S. oralis/mitis*	53	95	29	20	86	71
*S. sanguinis*	67	45	50	10	82	95
*S. salivarius*	23	35	31	10	32	38
*S. sobrinus*	39	80	43	70	34	33
*S. mutans*	30	70	43	85	68	5

100% indicates that all biofilm samples taken from a given volunteer, wire, enamel or saliva contain the indicated marker strain.
