# Supplementary material for: In vivo biofilm formation on stainless steel bonded retainers during different oral health-care regimens
Source: Int J Oral Sci. 2015 Jan 9;7(1):42–8. doi: 10.1038/ijos.2014.69 (PMC4817537; doi:10.1038/ijos.2014.69)
Supplement: Supplementary information [file ijos201469x1.doc]

**Supplementary information**

*In vivo* biofilm formation on stainless steel bonded-retainers

during different regimens of oral health care

Marije A. Jongsma^1^, Henny C. van der Mei^2^, Jelly Atema-Smit^2^, Henk J. Busscher^2^, Yjijn Ren^1^

^1^University of Groningen and University Medical Centre, Department of Orthodontics, Hanzeplein 1, 9700 RB Groningen, The Netherlands

^2^University of Groningen and University Medical Centre, Department of Biomedical Engineering, A. Deusinglaan 1, 9713 AV Groningen, The Netherlands

Running title: *In vivo* biofilm formation on bonded-retainers

Address for correspondence:

Prof. Dr. Y. Ren

Department of Orthodontics

University Medical Center Groningen

Hanzeplein 1, 9700 RB Groningen, The Netherlands

[y.ren@umcg.nl](mailto:y.ren@umcg.nl)

tel: +31(0)503610101

**Microbial adhesion to hydrocarbons (MATH)**

*S. mutans* ATCC 10449 grown on blood agar plates from a frozen stock, was used to inoculate 10 mL Tryptone Soya Broth (TSB) and cultured for 24 h at 37ºC. This culture was used to inoculate 100 mL TSB, which was grown overnight. Bacteria were harvested by centrifugation and washed twice with potassium phosphate buffer (pH 7.0) and suspended to an optical density *A_0_* (at 600 nm) of between 0.4 and 0.6. Next, half of the suspension was mixed with the supernatant of a toothpaste slurry (25% by weight) in water used after centrifugation, 5 min at 10,000 g to remove particulate matter for 2 min, centrifuged, washed and resuspended in potassium phosphate buffer to an optical density *A_0_* (at 600 nm) of between 0.4 and 0.6. In order to measure the hydrophobicity of the bacterial cell surfaces before and after exposure to a toothpaste supernatant, 150 µL hexadecane was added to 3 mL of each suspension and the suspension was vortexed for 10 s, allowed to settle for 10 min for phase separation and finally the optical density *A_t_* of the aqueous phase was measured. This was repeated 6 times and log (*A_t_/A_0_* x100) was plotted against the vortexing time (Figure S1). Initial removal rates R_0_ (min^-1^) were calculated as the slopes of the tangent of the curves obtained and used to compare effects of adsorption of toothpaste components on the hydrophobicity of the streptococcal cell surface (Table S1).

**Table S1.** Cell surface hydrophobicity of *S. mutans* ATCC 10449 before and after exposure to slurries of the different toothpastes involved in this study, as measured by the kinetic MATH assay and expressed in terms of their initial removal rates. All data represent the average ± SD of three experiments with separately grown bacteria.

| **Toothpaste used** | **Initial removal rate (min^-1^)** |
| --- | --- |
| *None* | 0.01 ± 0.01 |
| *Toothpaste without antibacterial claims* | 0.0 ± 0.0 |
| *Stannous fluoride containing toothpaste* | 0.0 ± 0.0 |
| *Triclosan containing toothpaste* | 0.05 ± 0.01^1^ |

^1^ Significantly different from all other data at *p<*0,000 (A One-Way ANOVA was used with a Bonferroni test for post-hoc multiple comparisons. Statistical significance was set at *p* < 0.05.

**
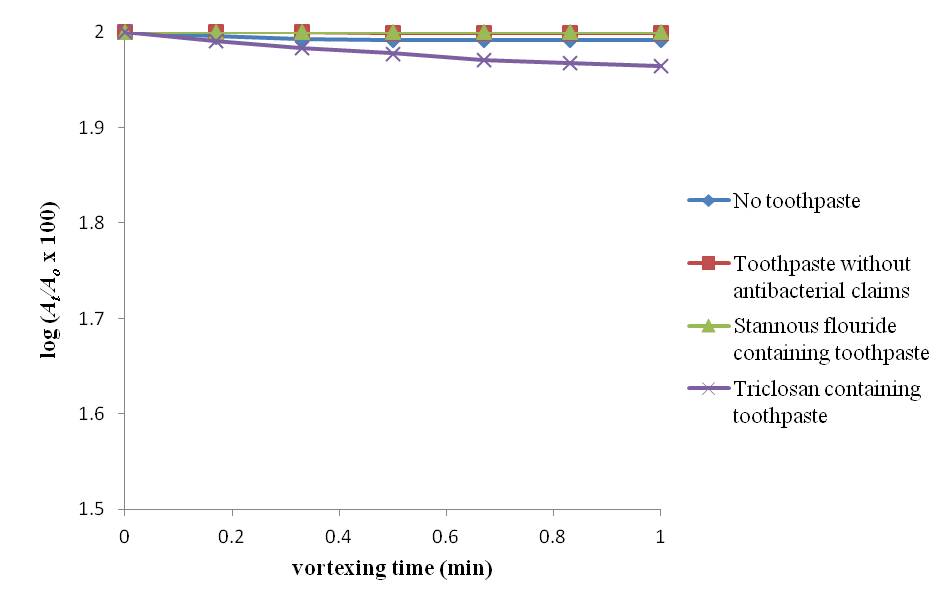
**

**Figure S1** Optical density log (*A_t_/A_0_* x100) as a function of the vortexing time for the removal of *S. mutans* ATCC 10449 prior to or after its exposure to a toothpaste slurry by hexadecane. Each data point represents the average over three experiments with different bacterial cultures. Standard deviations are smaller than the data points.
